# Anion-Binding-Induced Electrochemical Signal Transduction in Ferrocenylimidazolium: Combined Electrochemical Experimental and Theoretical Investigation

**DOI:** 10.3390/molecules24020238

**Published:** 2019-01-10

**Authors:** Tan-Qing Weng, Yi-Fan Huang, Lou-Sha Xue, Jie Cheng, Shan Jin, Sheng-Hua Liu, De-Yin Wu, George Z. Chen

**Affiliations:** 1 Key Laboratory of Pesticide & Chemical Biology of the Ministry of Education, College of Chemistry, Central China Normal University, Wuhan 430079, China; wengtq2007@gmail.com (T.-Q.W.); xuels2012@gmail.com (L.-S.X.); zhaohrt1983@gmail.com (J.C.); chshliu@mail.ccnu.edu.cn (S.-H.L.); 2 State Key Laboratory of Physical Chemistry of Solid Surfaces and Department of Chemistry, College of Chemistry and Chemical Engineering, Xiamen University, Xiamen 361005, China; yifanh@xmu.edu.cn (Y.-F.H.); dywu@xmu.edu.cn (D.-Y.W.); 3 Department of Chemical and Environmental Engineering, and Advanced Materials Research Group, Faculty of Engineering, University of Nottingham, Nottingham NG7 2RD, UK; George.Chen@nottingham.ac.uk

**Keywords:** electrostatic interactions, electron transfer, anion recognition, ferrocene alkylmethylimidazolium

## Abstract

Five ferrocene alkymethylimidazolium cations **1a**–**1d** and **2** with different alkyl spacer lengths were reinvestigated using voltammetry and density functional theory (DFT) calculations. The voltammetric responses of ligand **2** toward various anions are described in detail. An interesting and unprecedented finding from both experimental and theoretical studies is that coupled electron and intramolecular anion (F^−^) transfer may be present in these molecules. In addition, it was also observed that, in these studied molecules, the electrostatic attraction interaction toward F^−^ would effectively vanish beyond 1 nm, which was previously reported only for cations.

## 1. Introduction 

Non-covalent interactions are present in various modified and finely tuned forms in nature. As a primary example, electrostatic interactions play the decisive role in a range of intra- and intermolecular phenomena in the biological world [1,2,3]. Such interactions, depending on their strength and direction, could direct protein folding, binding, flexibility, stability, and functions. In addition, they are the main driving forces for signaling in biomacromolecules and supramolecules. Their influences exert on the host-guest combination, recognition, and dissociation processes in biological systems, for example, antigen-antibody recognition [1,2,3,4,5]. If the signal produced by antigen-antibody recognition can be detected by devices, diseases can be treated early and more effectively. Unfortunately, existing techniques and devices are not yet able to effectively and accurately detect such antigen-antibody recognition signals, hindering high-quality healthcare.

Host molecules combining both receptor and redox-active moieties attracted research interest continuously in the past three decades. In particular, various molecular devices, such as sensors, switches, shuttles, and logic gates, were proposed and demonstrated in recent years, mimicking how nature explores non-covalent interactions [4,5,6,7,8,9,10,11,12,13,14,15]. The redox moieties play the role of reporting intramolecular interactions for electrochemical analyses. Because of such a function, many efforts were made to combine one or more redox centers with receptor molecule receptors, aiming at electrochemical recognition of ionic guests. Anion recognition by redox-active receptor molecules is a topical area because of the important functions of anions in various chemical and biochemical processes and the relevant environments [9]. For electrochemical recognition of anions, redox-active receptor molecules were proposed and made to take advantage of communication through electrostatic, hydrogen-bonding, hydrophobic, coordinative, and/or conformational pathways [9]. In particular, electrostatic communications are perhaps the choice of high importance when designing redox-active receptor molecules for the electrochemical recognition of anionic guests, particularly in hydrophobic environments. Understanding of electrostatic interactions is important for not only understanding many naturally occurring biological processes, but also the design and fabrication of anion sensors. 

Receptor molecules with a ferrocene center are widely applied for electrochemical recognition and sensing, largely due to the reversible electro-oxidation of ferrocene to a ferrocenium ion that can exert strong electrostatic interactions [9,16,17,18,19,20]. On the other hand, anion binding by a host molecule is usually achieved or assisted via hydrogen bonding (H-bonding) with the H-donating amide or urea groups, [8,9,21,22,23,24], or H-accepting moieties, such as amine, pyridine, or bipyridine in the host molecules [8,9,21,22,23,24,25]. Ammonium- and guanidinium-containing hosts are important examples capable of offering strong electrostatic attractions to anionic guests [19,25,26,27,28]. In addition, receptors containing imidazolium [29,30,31,32,33,34,35,36,37] and triazolium [38,39] are able to recognize anions via a combined strong electrostatic attraction to anions and the (C–H)^+^···X^−^ type of H-bonding [25]. The charge transfer reaction of the redox center, e.g., ferrocene, can be affected in several ways by linking it with the receptor moieties, according to the type of guest anions and the linking bond [29,40,41,42]. 

**Scheme 1:**
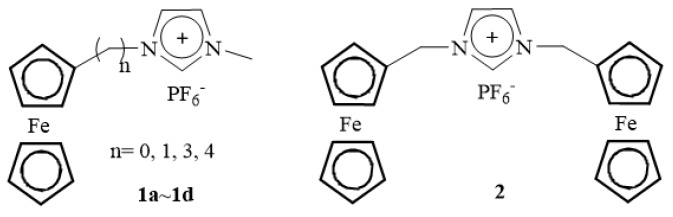
Schematic representations of the 1-ferrocene alkylmethylimidazolium salts, **1a**–**1d** (**left**, *n* = 0, 1, 3, and 4), and the di(ferrocenylmethyl)imidazolium salt **2** (**right**).

To focus the research effort on the understanding of electrostatic communications involving anionic guests, we propose to use a series of host molecules in which the ferrocene is linked to the imidazolium receptor via an alkyl chain of various lengths. It follows our previously reported findings that the electrostatic repulsions between the ferrocenium cation and the alkali metal cations that were hold in place by benzo-15-crown-5 agreed highly satisfactorily with the Coulomb Law, and that the observed interactions vanished when alkyl chain length was increased above 1 nm [43,44]. More recently, we published the synthetic and electrochemical details of a series of 1-ferrocene alkylmethylimidazolium cations **1a**–**1d** (Scheme 1), and demonstrated their anion binding electrochemical sensing properties, particularly for the F^−^ anion [33]. The present study did not aim to target a particular guest anion or attempt to tune any electrochemical response; rather, we hoped to investigate the electrostatic interaction exerted on the binding of guest anions through a reinvestigation of the behavior of the monoferrocene imidazolium salt. An interesting and unprecedented finding from combined electrochemical experimental results and density functional theory (DFT) calculations is that coupled electron and intramolecular anion (F^−^) transfer may be present in these molecules. More importantly, the study revealed that electrostatic interactions in anion-binding host molecules also effectively vanish when the alkyl spacer between the ferrocene and the anion-binding site is longer than 1 nm. 

## 2. Results 

### 2.1. Crystal Structures

**Figure 1:**
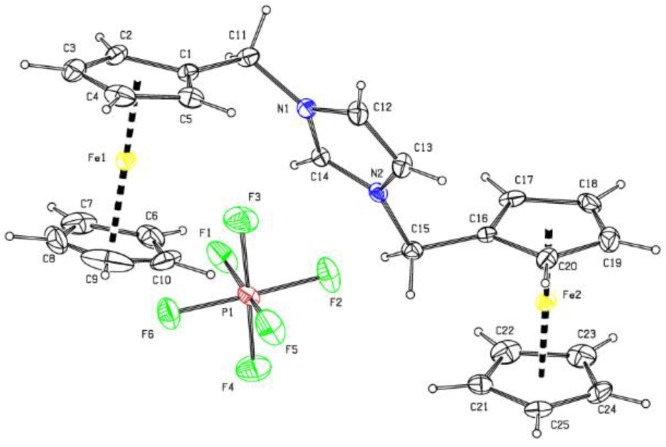
Molecular structure of di(ferrocenylmethyl)imidazolium salt **2**.

X-ray analysis of a single crystal of **2** was obtained via slow diffusion of diethyl ether into a saturated solution of acetone. We previously reported a single crystal structure of **1a** and **1b**, in which the imidazolium moieties were rotated out the plane of the Cp rings by about 37.8° and 89.4°, respectively [33]. As can be expected, receptor **2** exhibited the cisoid formation with the two ferrocenyl arms situated on the same side of the imidazolium moiety (Figure 1).

### 2.2. Electrochemical Behavior of the Ligand **2**

**Figure 2:**
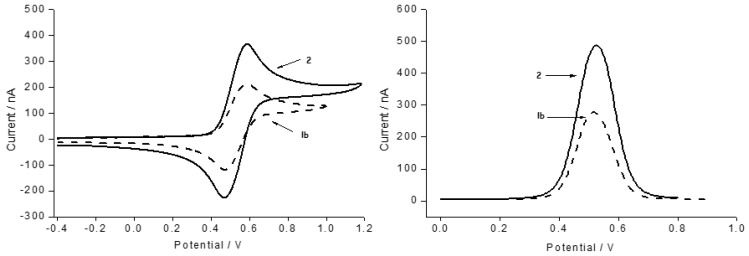
Cyclic voltammogram (CV) and squarewave voltammetry (SWV) response of free receptors **1b** and **2** (1 × 10^−3^ mol·dm^−3^) in CH_3_CN; supporting electrolyte: 0.1 M (n-Bu)_4_NB(C_6_F_5_)_4_; scan rate: 100 mV/s.

Similar to the behavior in ligands **1a**–**1d [33]**, cyclic voltammograms (CVs) of **2** (1 × 10^−3^ mol·dm^−3^) in dry acetonitrile exhibited a pair of well-defined reversible peaks at ca. 0.26 V (vs. Ag/Ag^+^). Table 1 lists the mean potential values of all five ligands measured against Ag/Ag^+^ or Fc/Fc^+^. It is noteworthy that oxidation of di(ferrocenylmethyl)imidazolium salt **2** and 1-ferrocene methylimidazolium salt **1b** occur at nearly the same potential, and display a single oxidation wave. The reversible two-electron oxidation can be assigned to the simultaneous one-electron oxidation of each of the two ferrocenyl moieties in **2** and the lack of electron communication between the two centers. As shown in Figure 2, this simultaneous two-electron redox process does not split into two separated one-electron events, even when a very low-coordinating electrolyte (e.g., Bu_4_NB(C_6_F_5_)_4_, tetra-*n*-butylammonium fluoride (TBAF)) and a lower-polarity solvent (e.g., CH_2_Cl_2_) were used [45]. Our findings contradict the results reported by Howarth et al., whereby receptor **2** shows two cathodic and two anodic waves [29]. This suggestion agrees with the fact that the two Fc moieties in **2** are sterically well separated by a non-conjugated –CH_2_– subunit. Further evidence comes from an analysis of the ^13^C-NMR spectral properties (not shown) and *E*_1/2_ of the two ferrocenyl substituents of **2**, which showed the typical pattern of regular monosubstituted ferrocenes **1b**, indicating no unusual charge distribution involving the adjacent metallocenyl moieties. This result also agrees well with previous reports that an electron-withdrawing group should be less favorable for electronic communication between their two metal termini [46,47]. 

It is interesting that the *E*_1/2_ values of these receptors actually reflect the distribution of the electrostatic repulsion between ferrocenium and the methyl imidazolium cation. In acetonitrile, it is known that each methyl substitute can negatively shift *E*_1/2_ of ferrocene by about 50 mV [18,20], and further addition of the –CH_2_– subunit to the alkyl chain may shift the potential by a value between −5 mV and −10 mV. Based on the potential shift induced by the electron-withdrawing effect of the methyl imidazolium cation subunits, one can estimate that the ∆*E*_1/2_ values of these ferrocene-based receptors should be about 324 mV, 217 mV, 53 mV, and 42 mV (vs. Fc/Fc^+^) for *n* = 0, 1, 3, and 4, respectively. Upon increasing the number and length of the methyl imidazolium cation spacers, the electrostatic repulsion effect was significantly reduced, and the *E*_1/2_ values of the alkyl-linked ferrocene-based receptors became more negative than that of the simple ferrocene.

**Figure 3:**
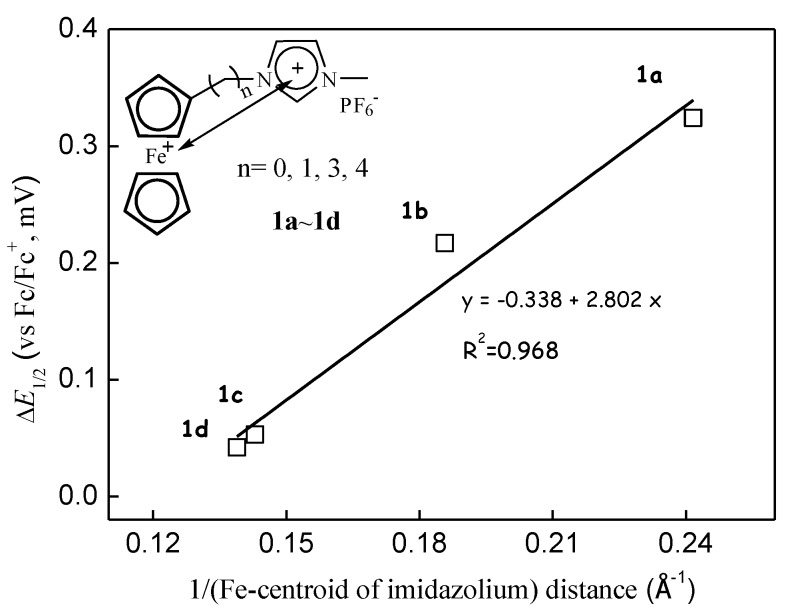
The mean potential of 1-ferrocene alkylmethylimidazolium salts **1a**–**1d** vs. Fe-centroid of imidazolium distance in the ligand.

Therefore, we sought to correlate redox potential with the Fe-centroid of imidazolium distance in the ligand. For **1a** and **1b**, these distances were taken directly from the X-ray data, and gave values of 4.138 Å for **1a** and 5.385 Å for **1b**. Due to the flexibility of the chain in the ferrocenes **1c** and **1d,** it is not possible to rely on X-ray data for the determination of the Fe-centroid of imidazolium distance; however, a density functional theory method gives a value of 6.994 Å for **1c** and 7.200 Å for **1d**. A good linear correlation (*R^2^* = 0.968) was found between the redox potentials and the Fe-centroid of imidazolium distance (Figure 3). As Δ*E*_1/2_ = 0, the value of *l*_max_ could be calculated from equation *y* = −0.338 + 2.802*x* given in Figure 3, and was found to be 0.80 ± 0.03 nm for ligands **1a**–**1d**. This relationship also confirms the results previously obtained, whereby electrostatic interactions would effectively vanish beyond a 1 nm spacer length [43].

### 2.3. Ligand **2** Electrochemical Recognition of Anions

Previously, we reported the electrochemical properties of monoferrocene methylimidazolium salt **1a**–**1d** toward various anions. In present study, the behavior of diferrocene imidazolium salt **2** in the presence of the selected anions F^−^, Cl^−^, Br^−^, and HSO_4_^−^ was also reinvestigated using CV and squarewave voltammetry (SWV) in CH_3_CN containing 0.1 mol·dm^−3^ Bu_4_NPF_6_. 

**Figure 4:**
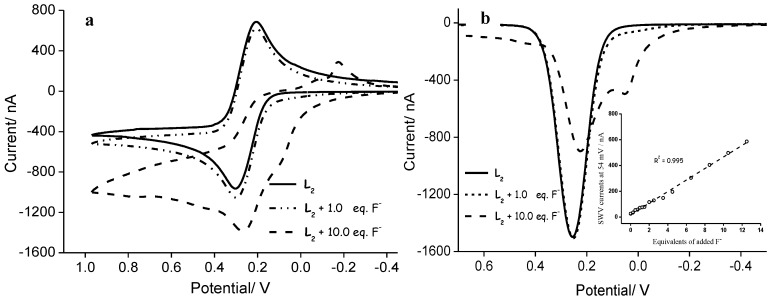
CV (**a**) and SWV (**b**) of receptor **2** in acetonitrile in the absence and presence of Bu_4_NF. Scan rate: 100 mV·s^−1^; ligand concentration close to 1.0 mmol·dm^−3^. The inset shows the variations of the SWV peak current of **2** with the addition of Bu_4_NF.

The basic voltammetric features of ligand **2** toward various anions are similar to that of ligand **1b**. Addition of anions to ligand **2** solution also showed two different behaviors: i.e., the “two-wave behavior” and the “single-wave behavior” [33]. As shown in Figure 4, when F^−^ was added, a shoulder was seen at the negative foot of the oxidation peak, which should be attributed to the formation of a **L_2_**·F^−^ complex, along with the appearance of a small reduction peak at ca. −0.2 V when 4.0 equivalents of F^−^ were added. The redox wave became fully irreversible and the shoulder reached full development when more than ten molar equivalents of F^−^ were added. A clear two-wave behavior was observed in SWV experiments (Figure 4b), with Δ*E*_p_ = *E*_p_^c^ − *E*_p_^r^ = −0.200 V (E_p_^c^ and E_p_^r^ are the peak potentials for the complexed and free receptor). This two-wave SWV behavior allowed an amperometric titration curve to be drawn by considering the intensities of the new peak I_p_ as a function of the F^−^/L**_2_** molar ratio (inset of Figure 4b). This curve displayed a good linear correlation between the peak current and the concentration of F^−^ ranging from 5.0 × 10^−5^ to 1.3 × 10^−2^ mol·dm^−3^.

**Figure 5:**
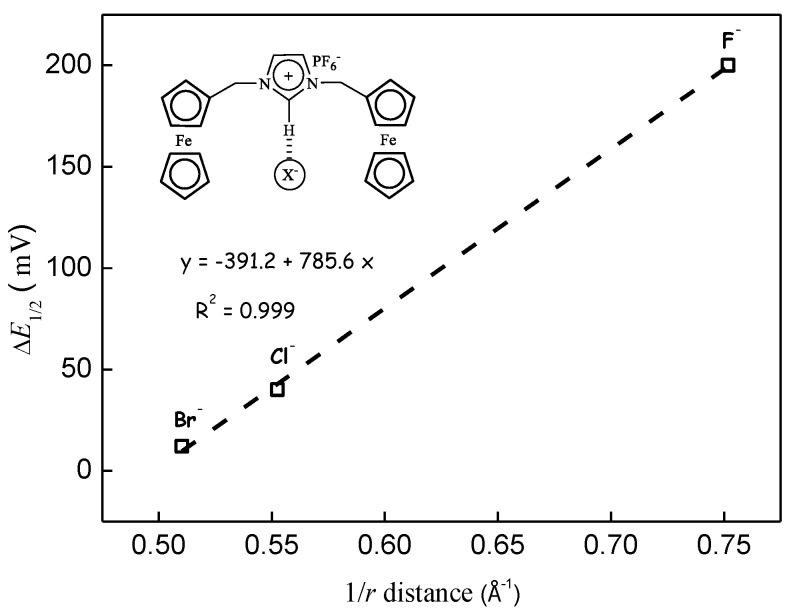
Anion-binding-induced potential shift as a function of the reciprocal of the radius of the halide ion in ligand **2**.

**Scheme 2:**
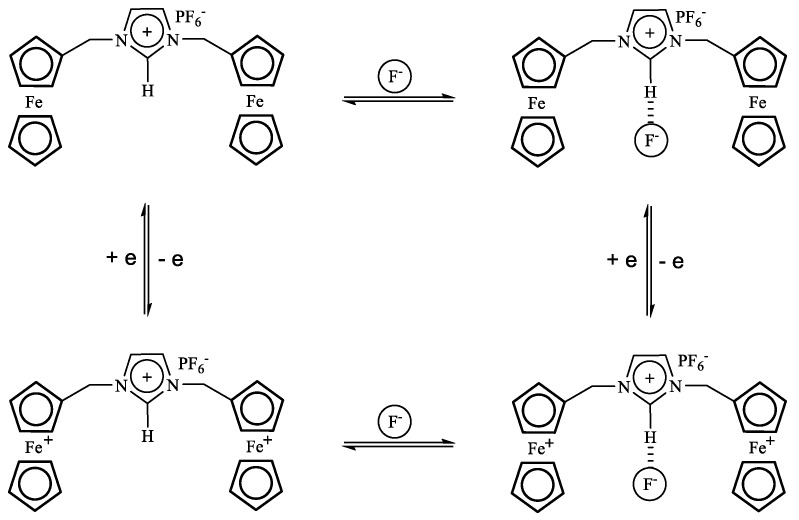
The scheme of one square for F^−^ binding and electron transfer.

In contrast, addition of other anions showed a “single-wave behavior”, i.e., the peak of the free receptor shifted to a slightly negative potential. The obtained Δ*E* values (vs Fc/Fc^+^) are listed in Table 2. As can be seen from Table 2, addition of F^−^ resulted in a very large cathodic shift of the Fc/Fc^+^. Addition of other anions also caused a small cathodic shift. The magnitude of the Δ*E*_p_ values follows the order F^−^ > Cl^−^ > Br^−^, which agrees with the negative charge density order of these anions. The reason that a slightly large potential shift happened in the case of ion pairs containing H_2_PO_4_^−^ and HSO_4_^−^ may be ascribed to the great differences in size and structure between the two anions and halogen anion. Therefore, we sought to determine linear relationships between the shifts of the mean potentials and the radius of halogen anions (*r*). As shown in Figure 5, a perfect linear correction (*R^2^* = 0.999) was found between Δ*E* and 1/*r*. On the basis of the X-ray crystal structure of the free ligand **L_2,_** rational conclusions could be drawn that the complex **L_2_**·X^−^ would form a cisoid formation with the halogen anions captive inside (Scheme 2), in which the anion would be in the middle between the two Fc^+^ centers, each of which exerts equal electrostatic attraction to the anion. 

## 3. Discussion

### 3.1. Coupled Electron and Inter- or Intramolecular Anion Transfer Processes

**Scheme 3:**
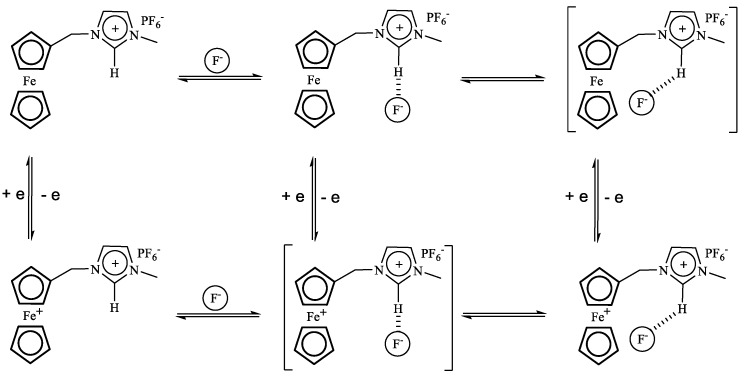
The scheme of electron transfer coupled with the F^−^ ion transfer processes in **1b.**

**Figure 6:**
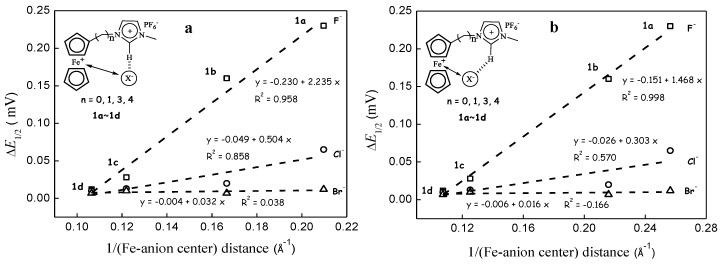
Complexation-induced potential shifts of **1a**–**1d** as a function of the inverse Fe–anion center distances when (**a**) neglecting and (**b**) considering the intramolecular anion transfer process.

Upon comparing between the shifts of the mean potential obtained with **2** and **1b** in the presence of Cl^−^ and Br^−^, the ratios of the Δ*E*_1/2_ values were close to 2, which can be seen as a reflection of the fact that each of the *bis*-substituted imidazolium ligands **2** had two Fc centers, while that of the mono-substituted **1b** had only one. If so, the formation of the complex **L_1b_**·F^−^ should have produced a negative potential shift by about 100 mV, approximately half of the potential shift for the complex **L_2_**·F^−^ (200 mV). In Figure 6a, the expected potential shifts of the ferrocene center are shown by the circular markers. Surprisingly, the measured ∆*E*_1/2_ values of the complex **L_1b_**·F^−^ (160 mV) were much larger than generally expected (100 mV). This suggests that there might be a stronger interaction between Fc^+^ and F^−^ in **L_1b_^+^**·F^−^ than what would be expected. However, because both ligands **1b** and **2** bind anions in their neutral states, as revealed by other imidazolium salts, it is likely that a bound anion may, in addition, form an ion pair with the Fc^+^ moiety, in the same way that a free anion can. Therefore, interesting conclusions may be drawn from this discrepancy. The electrochemical oxidation of Fc caused an intramolecular anion (F^−^) transfer in **L_1b_**·F^−^. We propose Scheme 3, where the electron transfer is coupled with the bound F^−^ ion transfer processes in **1b**.

To elucidate these coupled electron and intramolecular anion transfer processes, DFT calculations were carried out on the target systems **1a**–**1d** using the X3LYP/6-311+G** basis set. The geometries of a series of ferrocene alkymethylimidazolium cation complexes with different alkyl spacer lengths were fully optimized. For the complex **L_1_**·F^−^, the Fe–F^−^ distances were 4.768 Å for **1a**, 6.003 nm for **1b**, 8.200 Å for **1c**, and 9.395 Å for **1d**. However, for its oxidation state **L_1_^+^**·F^−^**,** the Fe–F^−^ distances were 3.900 Å for **1a**, 4.632 nm for **1b**, 7.990 Å for **1c**, and 9.318 Å for **1d**. It is obvious that, upon increasing the alkyl spacer length, intramolecular F^−^ transfer distance was greatly reduced after the removal of one electron in the ferrocene center. For example, for **1b**, intramolecular F^−^ transfer distances were close to 1.4 Å from the complex **L_1b_**·F^−^ to its oxidation state **L_1b_^+^**·F^−^, while only over a short distance for **1c** of about 0.21 Å. 

To further confirm these processes, we sought to correlate the redox potential shift with the Fe–F^−^ distance calculated in the two conformations. A good linear correlation (*R^2^* = 0.958) was found between the redox potential shifts Δ*E*_1/2_ and the Fe–F^−^ distance (*l*) by neglecting intramolecular F^−^ transfer (Figure 6a). However, as shown in Figure 6b, a perfect linear correlation (*R^2^* = 0.998), was obtained between the redox potential shifts and Fe–F^−^ distance (*l*) by considering intramolecular F^−^ transfer. This clearly indicates that coupled electrochemical oxidation and intramolecular anion (F^−^) transfer may occur particularly in short alkyl-linked methyl imidazolium salts **1a** and **1b**. With the decrease in alkyl length, the bound F^−^ resides spatially close to the ferrocenyl subunit, where an additional ion-pairing effect occurs; hence, the F^−^ ion exerts a much larger effect on the redox potential of the ferrocenyl group than would be predicted in terms of its high negative charge density, which suggests that the bound F^−^ resides closer to the ferrocenium moiety than any of the other halogen anions. However, for Cl^−^ or Br^−^, a very poor linear correlation was obtained between the redox potential shifts and Fe–X^−^ distance (*l*) by considering intramolecular anion transfer (Figure 6), which was also demonstrated when coupled electrochemical oxidation and intramolecular Cl^−^ or Br^−^ transfer processes might not be present in these molecular systems due to the low negative charge density. 

### 3.2. Through-Space Intramolecular Electrostatics

Of particular relevance to the intramolecular electrostatics are the shifts of the mean potential of receptors, Δ*E*_1/2_, induced by the anion binding. Assuming electrostatic interaction as the only intramolecular interaction between the receptors **1a**–**1d** and the bound anion, the following equation was derived previously to link Δ*E*_1/2_ with other parameters in Coulomb’s Law [43,44]:

(1) $$∆E_{1/2}=\frac{n∆Q_{Fc}Q_{anion}}{4\pi \epsilon_{0}\epsilon el}=\left(\frac{n∆Q_{Fc}}{4\pi \epsilon_{0}e}\right)\left(\frac{Q_{anion}}{\epsilon l}\right),$$

where *n* is the number of bound anions, ∆*Q_Fc_* is the variation in effective charge on the ferrocene moiety upon electron transfer, *l* is the distance between the bound anion and the ferrocene center, $\epsilon_{0}$ is the vacuum permittivity, ϵ is the the relative permittivity of the local medium between the bound anion and the ferrocene center, and *e* is the elementary charge. *Q_anion_* is Pauling’s definition of the effective nuclear charge, *ze*/*r_p_*, where *z* and *r_p_* are the bound anion’s valence and effective ionic radii [48]. For a halide ion, the effective ionic radii, which are 1.33 Å for F^−^, 1.81 Å for Cl^−^, and 1.96 Å for Br^−^, were used directly. Therefore,

(2) $$∆E_{1/2}=\left(\frac{n∆Q_{Fc}}{4\pi \epsilon_{0}e}\right)\left(\frac{ze}{\epsilon lr_{p}}\right).$$

**Figure 7:**
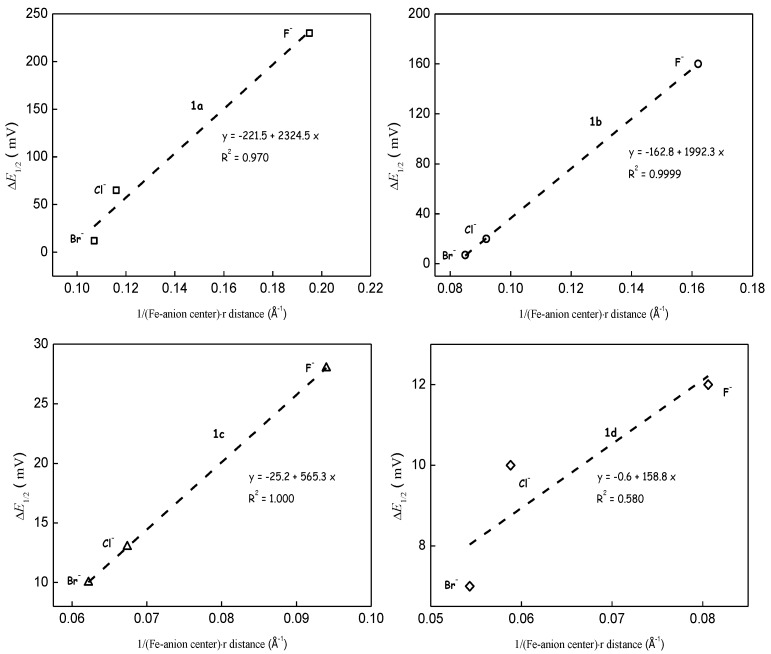
Anion-binding-induced potential shift as a function of the reciprocal of the Fe–anion distance and the radius of the anion when considering the intramolecular anion transfer process in ferrocene methylimidazolium receptor molecules **1a**–**1d**.

Equation (2) is also demonstrated by the good linear correlation between Δ*E*_1/2_ and the reciprocal of the *lr*_p_, as shown in Figure 7. 

The *l_max_* is the maximum interaction distance between the ferrocene center and the bound anion, at which the potential shift, ∆*E*_1/2_, drops to zero. In other words, beyond this length, the intramolecular electrostatic interactions are ineffective. Given ∆*E*_1/2_ = 0, the value of *l*_max_ can be calculated from the ratio of slope/intercept given in Figure 6, and was found to be 0.94 ± 0.05 nm for all anions [43]. This relationship demonstrated that through-space electrostatic attraction interactions would effectively vanish beyond a 1 nm spacer length for anion receptors toward anions. Previously, we also reported a similar result for the through-space electrostatic repulsion interaction in organic electrolytes of cation receptors toward cations, which became ineffective beyond a 1 nm spacer length. Therefore, on the basis of these relationships, a particularly interesting result can be drawn, whereby through-space intramolecular electrostatic interactions are ineffective beyond 1 nm.

## 4. Materials and Methods

### 4.1. Instrumentation

NMR spectra were measured using a Bruker AM300 spectrometer (Bruker Biospin GMBH, Rheinstetten, Germany) at 300 MHz for ^1^H and 75.45MHz for ^13^C. Column chromatography was performed on neutral alumina or silica. Electrochemical measurements were performed on a CHI660C potentiostat (CH Instruments Company, Austin, TX, USA). The ligand and electrolyte (Bu_4_NPF_6_ or Bu_4_NB(C_6_F_5_)_4_) concentrations were typically 0.001, 0.1, and 0.05 mol·dm^−3^. A three-electrode one-compartment cell was used, including 500-µm-diameter platinum-disc working electrodes, a platinum wire counter electrode, and an Ag|Ag^+^ reference electrode. 

### 4.2. Chemicals

CH_2_Cl_2_ was distilled from P_2_O_5_. Acetonitrile was pre-dried over class 4 Å molecular sieves (4–8 mesh) and then distilled from P_2_O_5_. Ferrocenylimidazolium salts **1a**–**1d** and **2** were prepared according to the literature procedures [29,33,49,50,51,52,53].

### 4.3. Crystallography

Crystals suitable for X-ray diffraction were grown from an acetone-diethyl ether solution. Intensity data were collected on a Nonius Kappa charge-coupled device (CCD) diffractometer with Mo Ka radiation (0.71073 Å) at 298 K. The structures were solved using a combination of direct methods (SHELXS-97 [54]) and Fourier difference techniques, and refined using full-matrix least-squares methods (SHELXL-2013 [55]) All non-H atoms were refined anisotropically. The hydrogen atoms were placed in the ideal positions and refined as riding atoms. Further crystal data and details of the data collection are summarized in Table S1 (Supplementary Materials). Selected bond distances and angles are given in Table S2 (Supplementary Materials).

### 4.4. Computational Details

DFT calculations were performed using Gaussian 09 package [56]. The geometries of a series of ferrocene alkymethylimidazolium cations with different alkyl spacer lengths were fully optimized and converged to the ground state by hybrid functional X3LYP, which better describes H-bonding (Figures S1 and S2, Supplementary Materials). The basis set for F, N, C, and H was 6-311+G**. The valence electron and core electron of Fe with the lowest spin were described by LanL2DZ and its effective core potential. In order to mimic the acetonitrile solution, a conductor-like polarizable continuum (CPCM) model was used in the optimization [57,58].

## 5. Conclusions

A series of alkyl-linked ferrocene imidazolium cations **1a**–**1d** and **2** were reinvestigated by voltammetry in the absence and presence of various anions in acetonitrile. An interesting derivation is that the electrochemical oxidation of the Fc center is coupled with an intramolecular F^−^ transfer from the imidazolium cation toward the Fc^+^ center in short alkyl-linked mono-ferrocene imidazolium cations. This is based on a comparison between the shifts of the mean potentials of **2** and **1b** upon addition of the halide ion, which was further confirmed by DFT calculations. A scheme linking the electron and bound F^−^ intramolecular transfer processes was proposed to account for the observed voltammetric phenomena. Anion-binding-induced mean potential shifts, ∆*E*_1/2_, were observed for receptors **1a**–**1d**, and found to vary linearly with the reciprocal of the Fe–anion distance, *l*, between the centers of the bound anion and the ferrocene in good accordance with Coulomb’s law. ∆*E*_1/2_ also increased linearly upon increasing the effective charge, *Q_anion_*, of the bound anion, represented by the charge-to-radius ratio, *ze*/*r*. A maximum alkyl spacer length of about 1 nm was extrapolated, beyond which the through-space electrostatic interactions were ineffective within the complex molecules in the electrochemical solution. This anion-induced effect agrees with the results shown with cations in previous studies. However, because anions play a more important role than cations in biological systems, this work will be more relevant to a better understanding of those biological processes involving intramolecular electrostatic interactions, e.g., protein folding and antigen-antibody recognition. Furthermore, the knowledge of a limited interaction distance for both cations and anions can offer greater flexibility for designing molecular devices to be operated by non-covalent forces. Finally, the findings presented in this work in relation with the effect of anions argue strongly for the previous prediction derived from the cation effect; in other words, the nanometer effect, which is well recognized when material dimensions decrease, is also true when molecule sizes increase.

## Figures and Tables

**Table 1:** Potential data of **1a**–**1d** and **2.**

Cation	1a	1b	1c	1d	2
*E*_1/2_ (vs. Ag/Ag^+^, mV)	413	256	72	56	262
*E*_1/2_ (vs. Fc/Fc^+^, mV)	324	167	−17	−33	173
Δ*E*_p_ (mV)	74	72	66	74	70
*i*_pa_/*i*_pc_	1.00	1.07	0.99	0.98	0.99

Mean potential: *E*_1/2_ = (*E*_pa_ + *E*_pc_)/2 (mV). All potential data, in mV, were determined in acetonitrile solutions containing ~1 × 10^−3^ mol·dm^−3^ compound and 0.1 mol·dm^−3^ Bu_4_NPF_6_ as the supporting electrolyte. The Ag|Ag^+^ electrode (internal solution: 0.01 mol·dm^−3^ AgNO_3_ + 0.10 mol·dm^−3^ Bu_4_NPF_6_ in acetonitrile; salt bridge: 0.10 mol·dm^−3^ Bu_4_NPF_6_ in acetonitrile) was used as a reference which reported a potential of 89 mV for the simple ferrocene|ferrocenium (Fc|Fc^+^) couple.

**Table 2:** Summary of the potential shifts measured in the presence of 10 molar equivalent of anions in acetonitrile solution.

Compound	Δ*E*_1/2_ (mV)			
	F^−^	Cl^−^	Br^−^	HSO_4_^−^
**1a**	230	65	12	108
**1b**	160	21	7	24
**1c**	28	13	10	24
**1d**	12	10	7	12
**2**	200	40	13	100

## Supplementary Materials

The Supplementary Materials are available online.

## References

[1] Kumar S., Nussinov R. (2002). Close-range electrostatic interactions in proteins. Chembiochem.

[2] Coleman J.A., Gouaux E. (2018). Structural basis for recognition of diverse antidepressants by the human serotonin transporter. Nat. Struct. Mol. Biol..

[3] Purohit A., England J.K., Douma L.G., Tondnevis F., Bloom L.B., Levitus M. (2017). Electrostatic Interactions at the Dimer Interface Stabilize the E. coli β Sliding Clamp. Biophys. J..

[4] Jeon W.S., Ziganshina A.Y., Lee J.W., Ko Y.H., Kang J.K., Lee C., Kim K. (2003). A [2]pseudorotaxane-based molecular machine: Reversible formation of a molecular loop driven by electrochemical and photochemical stimuli. Angew. Chem. Int. Ed..

[5] Janeway C.A., Travers P., Walport M., Shlomchik M.J. (2001). Immunobiology: The Immune System in Health and Disease, 5th ed.

[6] Jeon W.S., Kim E., Ko Y.H., Hwang I.H., Lee J.W., Kim S.Y., Kim H.J., Kim K. (2005). Molecular loop lock: A redox-driven molecular machine based on a host-stabilized charge-transfer complex. Angew. Chem. Int. Ed..

[7] Beer P.D., Gale P.A., Chen G.Z. (1999). Electrochemical molecular recognition: Pathways between complexation and signalling. J. Chem. Soc. Dalton Trans..

[8] Caltagirone C., Gale P.A. (2009). Anion receptor chemistry: Highlights from 2007. Chem. Soc. Rev..

[9] Molina P., Zapata F., Caballero A. (2017). Anion Recognition Strategies Based on Combined Noncovalent Interactions. Chem. Rev..

[10] Bdjic J.D., Balzani V., Credi A., Silvi S., Stoddart J.F. (2004). A Molecular Elevator. Science.

[11] Hawthorne M.F., Zink J.I., Skelton J.M., Bayer M.J., Liu C., Livshits E., Baer R., Neuhauser D. (2004). Electrical or photocontrol of the rotary motion of a metallacarborane. Science.

[12] Beer P.D., Gale P.A., Chen G.Z. (1999). Mechanisms of electrochemical recognition of cations, anions and neutral guest species by redox-active receptor molecules. Coord. Chem. Rev..

[13] Saleem M., Yu H., Wang L., Zain ul A., Khalid H., Akram M., Abbasi N.M., Chen Y. (2016). Study on synthesis of ferrocene-based boronic acid derivatives and their saccharides sensing properties. J. Electroanal. Chem..

[14] Fang Y., Zhou Y., Rui Q., Yao C. (2015). Rhodamine-Ferrocene Conjugate Chemosensor for Selectively Sensing Copper(II) with Multisignals: Chromaticity, Fluorescence, and Electrochemistry and Its Application in Living Cell Imaging. Organometallics.

[15] Caballero A., White N.G., Beer P.D. (2014). A ferrocene imidazolium-based macrocycle as an electrochemical chemosensor for halide anions. CrystEngComm.

[16] Beer P.D., Chen Z., Ogden M.I. (1995). Voltammetric and NMR studies of a bis(ferrocenecarboxamide)-substituted diaza 18-crown-6 receptor that simultaneously complexes and electrochemically recognises both cations and anions. J. Chem. Soc. Faraday Trans..

[17] Chen Z., Graydon A.R., Beer P.D. (1996). Electrochemical response to anions in acetonitrile by neutral molecular receptors containing ferrocene, amide and amine moieties. J. Chem. Soc., Faraday Trans..

[18] Goel A., Brennan N., Brady N., Kenny P.T.M. (2007). Electrochemical recognition of anions by 1,1′-*N*,*N*′ ferrocenoylbisamino acid esters. Biosens. Bioelectron..

[19] Reynes O., Bucher C., Moutet J.C., Royal G., Saint-Aman E. (2008). Electrochemical sensing of dihydrogen phosphate and adenosine-5′-triphosphate anions by self-assembled monolayers of (ferrocenylmethyl)trialkylammonium cations on gold electrodes. Inorg. Chim. Acta.

[20] Reynes O., Maillard F., Moutet J.C., Royal G., Saint-Aman E., Stanciu G., Dutasta J.P., Gosse I., Mulatier J.C. (2001). Complexation and electrochemical sensing of anions by amide-substituted ferrocenyl ligands. J. Organomet. Chem..

[21] Gale P.A., Quesada R. (2006). Anion coordination and anion-templated assembly: Highlights from 2002 to 2004. Coord. Chem. Rev..

[22] Gale P.A., Garcia-Garrido S.E., Garric J. (2008). Anion receptors based on organic frameworks: Highlights from 2005 and 2006. Coord. Chem. Rev..

[23] Mullen K.M., Beer P.D. (2009). Sulfate anion templation of macrocycles, capsules, interpenetrated and interlocked structures. Coord. Chem. Rev..

[24] Lorenzo A., Aller E., Molina P. (2009). Iminophosphorane-based synthesis of multinuclear ferrocenyl urea, thiourea and guanidine derivatives and exploration of their anion sensing properties. Tetrahedron.

[25] Yoon J., Kim S.K., Singh N.J., Kim K.S. (2006). Imidazolium receptors for the recognition of anions. Coord. Chem. Rev..

[26] Reynes O., Bucher C., Moutet J.C., Royal G., Saint-Aman E., Ungureanu E.M. (2005). Electrochemical sensing of anions by redox-active receptors built on the ferrocenyl cyclam framework. J. Electroanal. Chem..

[27] Reynes O., Royal G., Chainet E., Moutet J.C., Saint-Aman E. (2003). Poly(ferrocenylalkylammonium): A molecular electrode material for dihydrogenphosphate sensing. Electroanalysis.

[28] Bucher C., Devillers C.H., Moutet J.C., Royal G., Saint-Aman E. (2009). Ferrocene-appended porphyrins: Syntheses and properties. Coord. Chem. Rev..

[29] Thomas J.L., Howarth J., Kennedy A.M. (2002). Electrochemical anion recognition by novel ferrocenyl imidazole systems. Molecules.

[30] Xu Z., Kim S.K., Yoon J. (2010). Revisit to imidazolium receptors for the recognition of anions: Highlighted research during 2006–2009. Chem. Soc. Rev..

[31] Xu Z., Singh N.J., Kim S.K., Spring D.R., Kim K.S., Yoon J. (2011). Induction-Driven Stabilization of the Anion–π Interaction in Electron-Rich Aromatics as the Key to Fluoride Inclusion in Imidazolium-Cage Receptors. Chem. Eur. J..

[32] Xu Z., Kim S.K., Han S.J., Lee C., Kociok-Kohn G., James T.D., Yoon J. (2009). Ratiometric Fluorescence Sensing of Fluoride Ions by an Asymmetric Bidentate Receptor Containing a Boronic Acid and Imidazolium Group. Eur. J. Org. Chem..

[33] Kong D.D., Weng T.Q., He W.X., Liu B., Jin S., Hao X., Liu S.H. (2013). Synthesis, characterization, and electrochemical properties of ferrocenylimidazolium. J. Organomet. Chem..

[34] Su Z.-M., Yan X.-Q., Liang C.-L., Lin C.-X., Xie L.-L., Yuan Y.-F. (2016). Highly selective detection of fluoride based on 2,2-diferrocenylpropane benzimidazolium borate-ester salt. Tetrahedron Lett..

[35] Manivannan R., Elango K.P. (2015). Spectral and electrochemical studies on anion recognition by ferrocene based imidazoles possessing different electron acceptor moieties. J. Organomet. Chem..

[36] Zhuo J.-B., Lin C.-X., Wan Q., Xie L.-L., Yuan Y.-F. (2015). Anion receptors of N-ferrocenylmethylene-substituted bis-imidazolium salts linked by xylene spacers. J. Organomet. Chem..

[37] Zhuo J.-B., Zhu X.-X., Lin C.-X., Bai S., Xie L.-L., Yuan Y.-F. (2014). Design, synthesis and anion recognition of ferrocene-based benzimidazolium receptors. J. Organomet. Chem..

[38] Gilday L.C., White N.G., Beer P.D. (2012). Triazole- and triazolium-containing porphyrin-cages for optical anion sensing. Dalton Trans..

[39] Picot S.C., Mullaney B.R., Beer P.D. (2012). Ion-Pair Recognition by a Heteroditopic Triazole-Containing Receptor. Chem. Eur. J..

[40] Niu H.T., Yin Z.M., Su D.D., Niu D., Ao Y.B., He J.Q., Cheng J.P. (2008). Ferrocene-based imidazolium receptors for anions. Tetrahedron.

[41] Niu H.T., Yin Z.M., Su D.D., Niu D., He J.Q., Cheng J.P. (2008). Imidazolium-based macrocycles as multisignaling chemosensors for anions. Dalton Trans..

[42] Thomas J.L., Howarth J., Hanlon K., McGuirk D. (2000). Ferrocenyl imidazolium salts as a new class of anion receptors with C-H center dot center dot center dot X- hydrogen bonding. Tetrahedron Lett..

[43] Jin S., Wang D.H., Jin X.B., Chen G.Z. (2004). Intramolecular electrostatics: Coulomb’s law at sub-nanometers. Chemphyschem.

[44] Jin S., Jin X.B., Wang D.H., Cheng G.Z., Peng L., Chen G.Z. (2005). Voltammetric studies of through-space and through-bond electrostatic interactions in alkyl linked ferrocene and benzoaza-15-crown-5 receptor molecules in acetonitrile. J. Phys. Chem. B.

[45] Camire N., Mueller-Westerhoff U.T., Geiger W.E. (2001). Improved electrochemistry of multi-ferrocenyl compounds: Investigation of biferrocene, terferrocene, bis(fulvalene)diiron and diferrocenylethane in dichloromethane using [NBu_4_][B(C_6_F_5_)_4_] as supporting electrolyte. J. Organomet. Chem..

[46] Wu X.H., Weng T.Q., Jin S., Liang J.H., Guo R., Yu G.A., Liu S.H. (2009). Synthesis, characterization, and substituent effects of mononuclear ruthenium complexes [RuCl(CO)(PMe_3_)_3_ (CH=CH-C_6_H_4_-R-p)] (R = H, CH_3_, OCH_3_, NO_2_, NH_2_, NMe_2_). J. Organomet. Chem..

[47] Bard A.J., Faulkner L.R. (2001). Electrochemical Methods Fundamentals and Applications.

[48] Shannon R. (1976). Revised effective ionic radii and systematic studies of interatomic distances in halides and chalcogenides. Acta Crystallogr. A.

[49] Gao Y., Twamley B., Shreeve J.M. (2004). The first (ferrocenylmethyl)imidazollum and (ferrocenylmethyl)triazolium room temperature ionic liquids. Inorg. Chem..

[50] Coleman K.S., Turberville S., Pascu S.I., Green M.L.H. (2005). Synthesis of a new bidentate ferrocenyl N-heterocyclic carbene ligand precursor and the palladium (II) complex trans-[PdCl_2_(C^^^fc^^^C)], where (C^^^fc^^^C) = 1,1′-di-tert-butyl-3,3′(1,1′-dimethyleneferrocenyl)-diimidazol-2-ylidene. J. Organomet. Chem..

[51] Ozcubukcu S., Schmitt E., Leifert A., Bolm C. (2007). A general and efficient synthesis of nitrogen-substituted ferrocenes. Synthesis.

[52] Howarth J., James P., Ryan R. (2001). Sodium borohydride reduction of aldehydes and ketones in the recyclable ionic liquid [BMIM]PF_6_. Synth. Commun..

[53] Seo H., Kim B.Y., Lee J.H., Park H.J., Son S.U., Chung Y.K. (2003). Synthesis of chiral ferrocenyl imidazolium salts and their rhodium(I) and iridium(I) complexes. Organometallics.

[54] Sheldrick G.M. (1997). SHELXS-97, Program for Crystal Structure Solution.

[55] Sheldrick G.M. (2013). SHELXL-2013, Program for Crystal Structure Refinement.

[56] Frisch M.J., Trucks G.W., Schlegel H.B., Scuseria G.E., Robb M.A., Cheeseman J.R., Scalmani G., Barone V., Mennucci B., Petersson G.A. (2009). Gaussian 09, Revision A.02.

[57] Barone V., Cossi M. (1998). Quantum Calculation of Molecular Energies and Energy Gradients in Solution by a. Conductor Solvent Model. J. Phys. Chem. A..

[58] Cossi M., Rega N., Scalmani G., Barone V. (2003). Energies, structures, and electronic properties of molecules in solution with the C-PCM solvation model. J. Comput. Chem..

