# Supplementary material for: Anion-Binding-Induced Electrochemical Signal Transduction in Ferrocenylimidazolium: Combined Electrochemical Experimental and Theoretical Investigation
Source: Molecules. 2019 Jan 10;24(2):238. doi: 10.3390/molecules24020238 (PMC6359666; doi:10.3390/molecules24020238)
Supplement: Supplementary file 1 [file molecules-24-00238-s001.pdf]

Supporting Information

Anion Binding Induced Electrochemical Signal Transduction in Ferrocenylimidazolium --- Combined Electrochemical Experimental and Theoretical Investigation

Tan-Qing Weng[a], Yi-Fan Huang[b], Lou-Sha Xue[a], Jie Cheng[a], Shan Jin[a*], Sheng-Hua Liu[a], De-Yin Wu[b] and George Z. Chen [c]

[a] Key Laboratory of Pesticide & Chemical Biology of the Ministry of Education, College of Chemistry, Central China Normal University, Wuhan 430079, P. R. China

[b] State Key Laboratory of Physical Chemistry of Solid Surfaces and Department of Chemistry, College of Chemistry and Chemical Engineering, Xiamen University, Xiamen 361005, P. R. China

[c] Department of Chemical and Environmental Engineering, and Advanced Materials Research Group, Faculty of Engineering, University of Nottingham, Nottingham NG7 2RD, UK

1. **Additional synthetic procedures**

Di(ferrocenylmethyl)imidazolium iodide Under a nitrogen atmosphere, trimethylammoniometryferrocene iodide (2.90 g, 5.2 mmol) and imidazole (160 mg, 2.4 mmol) were added to dry DMF (10 mL), and the mixture solution was stirred and heated under reflux for 4 h. After cooling to room temperature, H_2O (50 mL) and Et_2O (50 mL) were added and the organic phase was separated. The aqueous phase was extracted with Et_2O until the organic phase became colorless. The combined organic phases were dried (Na_2SO_4), filtered and the solvent evaporated to give the crude product as yellow powder. The crude product was recrystallized from the mixture of CH_2Cl_2 and ether forming a yellow powder in 47% yield. ^{1}H NMR (600 MHz, CD_3COCD_3): δ (ppm) 4.23-4.26 (m, 14H, C5H4+ C5H5), 4.43 (d, J=2.4, 4H, C5H4), 5.31 (s, 4H, CH2), 7.01 (d, J=1.2, 2H, CH=CH), 10.20 (s, 1H, NCH=N).

Di(ferrocenylmethyl)imidazolium hexafluorophosphate A solid of NH_4PF_6 (163 mg, 1 mmol) was added to 20 mL of ethanol solution of *Di(ferrocenylmethyl)imidazolium iodide* (530 mg, 0.90 mmol), and the solution

was stirred for 24 h at room temperature. The mixture was extracted with CH_2Cl_2 and the solvent evaporated to give the crude product as yellow powder. The crude product was recrystallized from the mixture of acetone and ether as yellow needles in 65% yield. ^1H NMR (600 MHz, CD_3COCD_3): δ (ppm) 4.20-4.26 (m, 14H, C5H4+ C5H5), 4.35 (t, J=2.7, 4H, C5H4), 5.12 (s, 4H, CH2), 7.05 (d, J=1.8, 2H, CH=CH), 8.53 (s, 1H, NCH=N) ppm. ^{13}C NMR (100 MHz, CDCl3): δ (ppm) 49.76 (CH2), 69.14, 69.44, 69.62, 78.22 (Cp-C), 121.23, 133.72 (imidazole-C). ESI-MS: m/z 610.36 $(M\text{-}PF_6)^+$. Anal. Calcd for $C_{25}H_{25}F_6Fe_2N_2P$: C, 49.21; H, 4.13; N, 4.59%. Found: C, 49.02; H, 4.01; N, 4.65%.

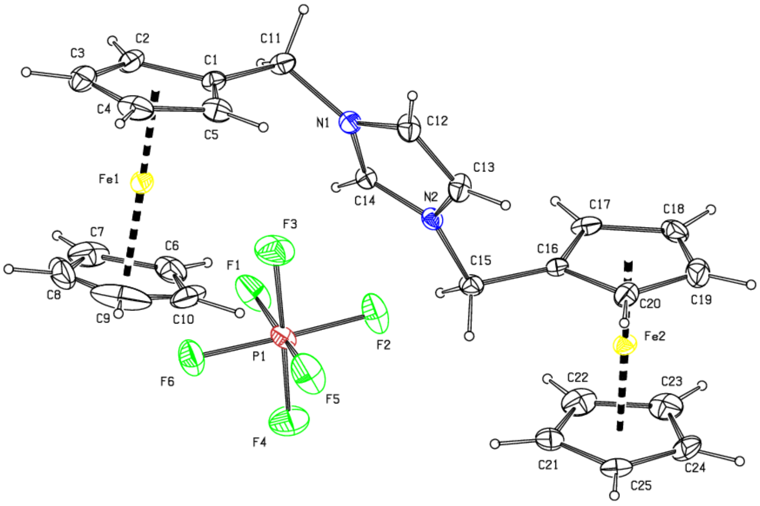

Table S1. X-ray crystallographic data of complex **2**.

**Compound**	**2**
Empirical formula	$C_{25}H_{25}F_6Fe_2N_2P$
Formula weight	610.14
Temperature(K)	298(2)
Wavelength(Å)	0.71073
Crystal system	Monoclinic
Space group	P2(1)/c
a(Å)	15.1872(5)
b(Å)	10.1702(4)
c(Å)	17.5415(6)
α(°)	90.00
β(°)	2.793(2)
γ(°)	90.00
Volume(Å^3)	2497.83(15)
Z	4
Density (calculated)(Mg/m^3)	1.622
F(000)	1240
Crystal size(mm^3)	0.23 x 0.10 x 0.10
θ(min-max)(°)	2.37 to 25.50
Reflections collected	24267
Independent reflections	4616
R(int)	0.0865
Refinement method	Full-matrix least-squares on F^2
Goodness-of-fit on F^2	1.091
R indices [I>2σ]	R_1 = 0.0596, ωR_2 = 0.1713
R indices (all data)	R_1 = 0.0721, ωR_2 = 0.1798

Table S2. Selected Bond Lengths (Å) and Angles (deg) for **2.**

Bonds	[Å]	Bonds	[Å]
C(1)-C(5)	1.414(5)	C(14)-N(1)	1.313(4)
C(1)-C(2)	1.427(6)	C(14)-N(2)	1.318(4)
C(1)-C(11)	1.490(5)	C(15)-C(16)	1.470(5)
C(2)-C(3)	1.437(7)	C(15)-N(2)	1.497(4)
C(3)-C(4)	1.405(8)	C(16)-C(17)	1.403(5)
C(4)-C(5)	1.416(7)	C(16)-C(20)	1.430(5)
C(6)-C(10)	1.307(9)	C(17)-C(18)	1.446(7)
C(6)-C(7)	1.366(10)	C(18)-C(19)	1.379(7)
C(7)-C(8)	1.380(12)	C(19)-C(20)	1.393(7)
C(8)-C(9)	1.230(15)	C(21)-C(25)	1.388(7)
C(9)-C(10)	1.308(13)	C(21)-C(22)	1.392(7)
C(11)-N(1)	1.464(5)	C(22)-C(23)	1.420(8)
C(12)-C(13)	1.329(5)	C(23)-C(24)	1.371(8)
C(12)-N(1)	1.374(5)	C(24)-C(25)	1.383(7)
C(13)-N(2)	1.356(4)		
**Angles**	**[°]**	**Angles**	**[°]**
C(5)-C(1)-C(2)	109.1(4)	C(17)-C(16)-C(20)	107.8(4)
C(5)-C(1)-C(11)	129.2(4)	C(17)-C(16)-C(15)	127.5(4)
C(2)-C(1)-C(11)	121.4(4)	C(20)-C(16)-C(15)	124.6(3)
C(1)-C(2)-C(3)	106.8(4)	C(19)-C(18)-C(17)	109.7(4)
C(4)-C(3)-C(2)	107.7(4)	C(18)-C(19)-C(20)	107.6(4)
C(3)-C(4)-C(5)	109.5(4)	C(19)-C(20)-C(16)	108.9(4)
C(1)-C(5)-C(4)	107.0(4)	C(25)-C(21)-C(22)	107.9(5)
C(10)-C(6)-C(7)	106.7(6)	C(21)-C(22)-C(23)	107.9(5)
C(6)-C(7)-C(8)	104.5(6)	C(24)-C(23)-C(22)	106.5(5)
C(9)-C(8)-C(7)	109.0(8)	C(23)-C(24)-C(25)	110.0(5)
C(8)-C(9)-C(10)	110.7(9)	C(24)-C(25)-C(21)	107.7(5)
C(6)-C(10)-C(9)	109.0(8)	C(14)-N(1)-C(12)	107.8(3)
N(1)-C(11)-C(1)	113.9(3)	C(14)-N(1)-C(11)	124.6(3)
C(13)-C(12)-N(1)	107.6(3)	C(12)-N(1)-C(11)	127.5(3)
C(12)-C(13)-N(2)	106.9(3)	C(14)-N(2)-C(13)	109.0(3)
N(1)-C(14)-N(2)	108.7(3)	C(14)-N(2)-C(15)	124.2(3)
C(16)-C(15)-N(2)	112.7(3)	C(13)-N(2)-C(15)	126.7(3)

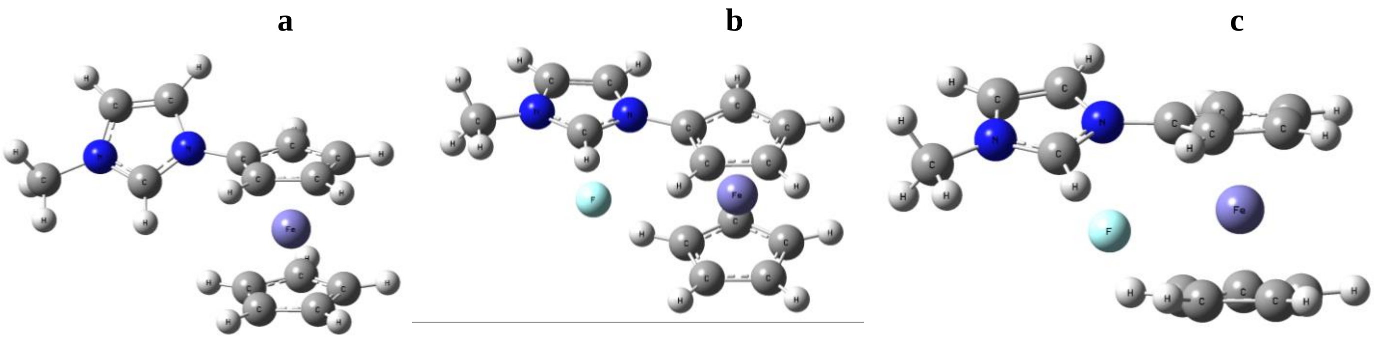

Figure S1. The molecular structure of (a) **1a**, (b) **1a**·F^{-} and (c) **1a**$^{+}$·F^{-} .

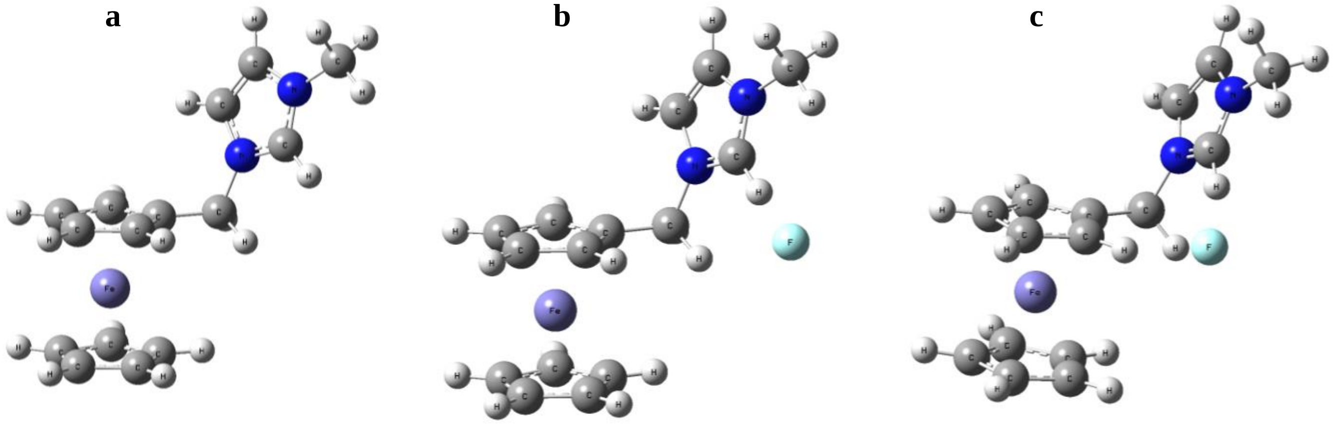

Figure S2. The molecular structure of (a) **1b**, (b) **1b**·F^{-} and (c) **1b**$^{+}$·F^{-}
